# Analysis of omission of antimicrobial doses in Intensive Care Units

**DOI:** 10.1590/0034-7167-2024-0102

**Published:** 2024-12-16

**Authors:** Isabelle Ribeiro Machado, Danielle de Mendonça Henrique, Flávia Giron Camerini, Vanessa Galdino de Paula, Cintia Silva Fassarella, Lucas Rodrigo Garcia de Mello

**Affiliations:** I Universidade do Estado do Rio de Janeiro. Rio de Janeiro, Rio de Janeiro, Brazil

**Keywords:** Anti-Infective Agents, Antimicrobial Stewardship, Intensive Care Units, Medication Errors, Patient Safety., Antiinfecciosos, Programas de Optimización del Uso de los Antimicrobianos, Unidades de Cuidados Intensivos, Errores de Medicación, Seguridad del Paciente.

## Abstract

**Objectives:**

to analyze the rate of antimicrobial dose omission in intensive care units.

**Methods:**

cross-sectional study carried out between March 1 and September 30, 2023, in intensive care units of a University Hospital in Rio de Janeiro.

**Results:**

the sample consisted of 452 prescriptions and 1467 antimicrobial doses. The dose omission rate was 4.29%. Each antimicrobial prescribed increased the chance of omission by 51%. The strategy of double-checking prescriptions helped prevent 30% of antimicrobial dose omissions (p=0.0001).

**Conclusions:**

monitoring the omission of antimicrobial doses can guide nursing actions to improve quality and patient safety, contributing to the prevention of medication errors, antimicrobial stewardship and the fight against antimicrobial resistance.

## RESUMEN

**Objetivos:**

analizar la tasa de omisión de dosis de antimicrobianos en unidades de cuidados intensivos.

**Métodos:**

estudio transversal, realizado entre el 1 de marzo y el 30 de septiembre de 2023, en unidades de cuidados intensivos de un Hospital Universitario de Río de Janeiro.

**Resultados:**

la muestra fue de 452 prescripciones y 1467 dosis de antimicrobianos. La tasa de omisión de dosis correspondió al 4,29%. Cada antimicrobiano prescrito aumentó la probabilidad de omisión en un 51%. La estrategia de doble comprobación de las prescripciones contribuyó a la prevención del 30% de las omisiones de dosis de antimicrobianos (p=0,0001).

**Conclusiones:**

el monitoreo de la omisión de dosis de antimicrobianos puede orientar acciones de enfermería para mejorar la calidad y la seguridad del paciente, contribuyendo a la prevención de errores de medicación, gestión de antimicrobianos y combate a la resistencia antimicrobiana.

## INTRODUCTION

Antimicrobials are the second most widely used class of drugs in hospitals and account for 20 to 50% of hospital drug expenditures^(1)^. In Latin America, it is estimated that antimicrobials are used inappropriately in 50% of cases, and Brazil leads the ranking of antibiotic consumption, with 22.75 daily doses defined for every thousand inhabitants per day^(2)^.

The indiscriminate usage of antimicrobials contributes to Antimicrobial Resistance (AMR), defined as the ability of a microorganism to prevent the action of an antimicrobial^(3)^. Infection by resistant organisms increases mortality, duration of illness, length of hospital stay and consequently has a very high cost^(4)^.

Intensive Care Units (ICUs) have been considered the epicenter of antimicrobial-resistant bacteria. They have faced major challenges in combating AMR, given the diversity and spread of microorganisms, due to contributing factors such as the use of invasive devices, immunosuppression, age, and empirical treatment with antibiotics and inappropriate use, as well as the incidence of Healthcare-Associated Infections (HAIs)^(4)^.

Medication errors related to antibiotic therapy are a problem in the ICU, which is exacerbated by aspects related to the weakness of pharmacology knowledge and inadequate work processes^(5)^. Omission of doses is a relevant medication error in the ICU, due to its frequent occurrence and potential harm to the patient. It is characterized by the failure to administer a necessary medication to the patient, or one or more prescribed doses until the next scheduled time, excluding circumstances in which the patient refuses to take the medication or when there is a medical decision not to administer it^(6)^. A British study that analyzed 90,761 doses of antimicrobials showed a dose omission rate of 7.2%, with 0.9% of the omitted doses being unavailable medications^(7)^. In Brazil, a study conducted in the ICU of a teaching hospital in the Federal District collected a sample of 711 doses of antimicrobials and 48 doses were omitted, corresponding to an error rate in the administration of antimicrobials due to dose omission of 6.75%^(8)^.

Patients admitted to the ICU are five to ten times more likely to develop HAIs^(9)^. This reflects antimicrobial polypharmacy, with prescriptions for latest-generation antimicrobials, contributing to irrational use and consequent antimicrobial resistance, which may increase the risk of omission of doses of an antimicrobial, causing harm to patient safety^(10)^.

A study in Ethiopia identified the prescription of antimicrobials in 80% of patients admitted to wards and 100% of patients in the ICU. However, less than 1% of these patients underwent microbiological tests before starting treatment, for an accurate diagnosis of infection by pathogenic microorganisms, corroborating the irrational use of antimicrobials and antimicrobial resistance^(11)^.

Antimicrobial management has been a fundamental strategy to mitigate ADR and dose omission^(12)^. It involves a set of actions aimed at controlling the use of these medications from diagnosis, selection, prescription and adequate dispensing to good dilution, storage and administration practices, in addition to auditing and monitoring of prescriptions, education of professionals and patients, monitoring of the program and adoption of interventionist measures, ensuring optimal therapeutic results with minimal potential risk^(13)^.

The effective participation of nurses in antimicrobial management programs, among other actions, can contribute to mitigating the omission of antimicrobial doses and reducing unfavorable outcomes such as overall and specific mortality rates related to multidrug-resistant microorganisms, length of hospital stay, incidence of HAIs, readmission related to infectious diagnoses and rates of adverse reactions to antibiotics. Reflecting in the improvement of quality of care and patient safety^(12)^.

## OBJECTIVES

To analyze the rate of antimicrobial dose omission in intensive care units.

## METHODS

### Ethical aspects

The study was conducted in accordance with national and international ethics guidelines and approved by the Research Ethics Committee (CEP) of the State University of Rio de Janeiro (UERJ), in accordance with Resolution 466/12, whose opinion is attached to this submission.

The Informed Consent Form (ICF) was waived from the beginning of data collection and survey was performed by accessing documents such as medical records and prescriptions using the registration number, which serves only to validate the individuality of the information. In addition, the confidentiality of the patients’ personal identification was guaranteed by the main investigator.

### Study design, period and location

This is a cross-sectional study, following the twenty-two steps of the Strengthening the Reporting of Observational Studies in Epidemiology (STROBE) recommendations^(14)^.

The study setting consisted of a general intensive care unit and a cardio-intensive care unit of a University Hospital of the Unified Health System (SUS), located in the city of Rio de Janeiro - RJ. Each unit has 10 inpatient beds and often maintains an occupancy rate of 100%. Data collection was carried out between March 1^st^ and September 30^th^, 2023.

### Population, sample, inclusion and exclusion criteria

The study population consisted of prescriptions from patients admitted to the two intensive care units over a 12-month period, totaling 7,920 prescriptions. To calculate the probabilistic sample, a simple random sample was chosen, in which all elements of the population have an equal probability of belonging to the sample, performed by the Epiinfo^®^ calculator. Considering the population described, this study chose to attribute as a maximum percentage the prevalence of 47.9% of errors related to dose omission described by the Institute for Safe Medication Practices (ISMP)^(5)^. A 97% confidence interval was attributed, thus the calculated sample was 444 prescriptions. The inclusion criteria were prescriptions from patients over 18 years old admitted to intensive care units, with an intravenous antimicrobial prescription. Prescriptions in which the doses were omitted due to patient refusal or medical suspension were excluded, since these cases do not characterize dose omission.

### Study protocol

The information of interest was collected from printed prescriptions and nursing records and electronic medical records, using a form whose variables were anchored in the ISMP Medication Error Prevention by Omission bulletin^(5)^ and entered into the Epimed Patient Safety Monitor^®^ software for incident management (Epimed Solutions^®^, Rio de Janeiro) that is integrated with the MV Soul^®^ System (electronic medical record used at the study hospital).

The variables related to the patient’s clinical profile were included: age, gender, diagnosis, date of admission and outcome (discharge, death and transfer), predictors of severity and mortality. In addition, variables related to dose omission were collected from medical prescriptions from the day before collection, total number of doses of antimicrobials prescribed and double checking.

It is noteworthy that in this study, dose omission was computed based on the analysis of prescriptions and nursing records of non-administration of the antimicrobial, in which doses that were not checked or were circled without justification were considered omitted. Furthermore, the study units adopt double checking as a medication safety tool, which can be audited, since the nurse records on the prescription that the double checking was performed and includes the date and stamp.

### Analysis of results and statistics

The data were organized and tabulated in a spreadsheet (Microsoft Excel^®^). Numerical variables were described as mean, standard deviation, median, first and third quartiles, and categorical variables as absolute and relative frequency. The dose omission rate indicator was calculated by dividing the number of omitted doses by the total number of antimicrobial doses prescribed.

The relationship between the omission rate and the variables of interest (place of hospitalization, gender, double checking, comorbidities, age, number of antibiotics, and the Charlson and Simplified Acute Physiology Score 3 (SAPS3) predictors of severity and mortality) was assessed using logistic regression models. 95% confidence intervals were calculated for the omission rates and estimated odds ratios. All analyses were conducted using the R statistical package, version 4.3.1.

## RESULTS

A total of 452 prescriptions from 94 patients admitted to intensive care units were analyzed. The dose omission rate calculated in this study was 4.29%, considering a total of 1,467 doses of antimicrobials analyzed and 63 omitted doses, that is, approximately 4 doses of antimicrobials were omitted in every 100 prescriptions.

The average number of antimicrobial doses prescribed per patient was 15.61 and the median was 7.5. The average age of the patients was 59 years, with the first quartile being 54 years and the third quartile being 69 years. This shows that half of the subjects are concentrated in this age group.

Table 1 shows the probability of antimicrobial dose omission according to the variables related to the patients. The results showed statistical significance, with p-values lower than 0.05 regarding the place of hospitalization, gender and the presence of comorbidities, cancer, hypertension, obesity, immunosuppression, congestive heart failure (CHF).

**Table 1:** Probability of omission of antimicrobial doses related to patient characteristics, Rio de Janeiro, Rio de Janeiro, Brazil, 2023

Predictor	Level	Prevalence	Probability	CI	*p* value
Place of Admission	General ICU	51.06%	5.39%	(4.11 - 7.05)	0.026
	Cardiointensive Care	48.94%	2.96%	(1.82 - 4.78)	
Gender	Female	48.94%	3.12%	(2.06 - 4.69)	0.013
	Male	51.06%	5.78%	(4.33 - 7.68)	
Comorbidities					
Cancer	No	72.34%	2.60%	(1.76 - 3.81)	<0.001
	Yes	27.66%	8.13%	(6.05 - 10.86)	
DM	No	41.49%	5.02%	(3.66 - 6.85)	0.332
	Yes	58.51%	3.97%	(2.77 - 5.66)	
Hypertension	No	24.47%	7.61%	(5.48 - 10.46)	<0.001
	Yes	75.53%	3.14%	(2.23 - 4.40)	
Obesity	No	94.68%	4.73%	(3.73 - 5.99)	0.049
	Yes	5.32%	1.06%	(0.15 - 7.16)	
Immunosuppression	No	71.28%	5.32%	(4.12 -6.85)	0.01
	Yes	28.72%	2.41%	(1.30 - 4.42)	
Asthma	No	93.62%	4.64%	(3.65 - 5.87)	0.17
	Yes	6.38%	1.52%	(0.21 - 9.98)	
CHF	No	56.38%	6.23%	(4.81 - 8.03)	<0.001
	Yes	43.62%	1.88%	(1.05 - 3.37)	
Chronic Atrial Fibrillation	No	78.26%	4.75%	(3.67 - 6.14)	0.644
	Yes	21.74%	4.10%	(2.29 - 7.26)	

*CI - 95% Confidence Interval.*

Regarding the place of hospitalization, 48 patients (51.06%) were hospitalized in the general intensive care unit and 46 (48.94%) in the cardio-intensive unit. It was observed that the general ICU presented a 5.39% probability of occurrence of omission of antimicrobial doses (p-value: 0.026). There was a balance in the prevalence of females 46 (48.94%) and males 48 (51.06%), but males were more likely to omit doses (5.78%) than females (3.12%). Among the comorbidities with the highest prevalence are systemic arterial hypertension (75.53%), diabetes mellitus (58.51%) and congestive heart failure (43.63%). However, in diabetic patients there was no significance to distinguish the rates evaluated. The prevalence of cancer was 27.66% and patients with this disease stood out for having a higher probability of omitting antimicrobial doses (8.13%) with relevant statistical significance (p-value <0.001).

Table 2 shows the probability of omitting doses based on the Charlson and Simplified Acute Physiology Score 3 (SAPS3) predictors and the amount of antimicrobials prescribed, representing aspects of the severity of the intensive care patient.

**Table 2:** Probability of occurrence of omission of antimicrobial doses in critically ill patients, Rio de Janeiro, Rio de Janeiro, Brazil, 2023

Predictors of severity	PR	CI	*p* value
*Charlson*	1.05	(0.92-1.18)	0.44
*SAPS3*	0.99	(0.97-1.01)	0.60
Number of Antimicrobials prescribed	1.51	(1.21-1.87)	<0.001

*PR - Prevalence Ratio; CI - 95% Confidence interval.*

Statistical significance (p<0.001) was identified regarding the quantity of antimicrobials per prescription. Each antimicrobial prescribed increases the chance of omission by 51%. Regarding the predictors of severity, although not significant, it can be observed that each unit of the Charlson comorbidity score increases the chance of omission of antimicrobial doses by 5%.

Table 3 shows the probability of occurrence of omission of antimicrobial doses due to the performance of double checking, a patient safety strategy performed by nurses aimed at preventing medication errors and antimicrobial management.

**Table 3:** Association of antimicrobial dose omissions with the double-check prescription safety strategy, Rio de Janeiro, Rio de Janeiro, Brazil, 2023

		Omission of Doses		PR	CI	*p* value
Exposure		Yes	No			
Double Check	Yes	11	249	0.30	0.15-0.59	0.0001
	No	27	165			
Total		38	414			

*PR - Prevalence Ratio; CI - 95% Confidence interval.*

Double checking was performed on 260 prescriptions and 11 of them showed omission of antimicrobial doses, with statistical significance (p value 0.0001). When calculating the prevalence ratio, it was shown that double checking can prevent omission of antimicrobial doses by 30%.

## DISCUSSION

Antimicrobial dose omissions are medication errors that have an unfavorable impact on critically ill patients and are considered an incident that results in increased length of hospital stay, risk of sepsis, and mortality^(15)^. In addition, antimicrobials are administered in relatively short cycles, and omitted doses can lead to high concentrations and contribute to antimicrobial resistance^(16)^.

Omission rates reported by previous studies are highly variable, possibly due to the different definitions and classification systems for omission. Therefore, it was difficult to draw comparisons, since some studies analyze “doses” and others “patients”. However, the present study revealed an antimicrobial dose omission rate of 4.29%. Another national study conducted in an intensive care unit analyzed a sample of 711 antimicrobial doses, and 48 omissions occurred, corresponding to an omission rate of 6.75%^(7)^. Other studies on the subject ranged from 0.9% in the United Kingdom to 28.8% in Zambia^(8,17)^. In addition to the rate of dose omissions, the characteristics of patient groups can be highlighted to be prioritized in strategies to prevent antimicrobial dose omissions^(17)^. When associating the characteristics of critically ill patients using antimicrobials with the omission of antimicrobial doses, this study observed a higher proportion of antimicrobial dose omissions in male patients (48.94% vs. 51.06%, [p=0.013]). This finding is in line with another study that also identified a higher prevalence of omissions in males (51.4% vs. 35.9%, [p=0.075])^(18)^. The most prevalent comorbidities with a higher probability of antimicrobial dose omission were among patients with systemic arterial hypertension, congestive heart failure, and cancer.

Considering this important epidemiological data, a study on medication discrepancy conducted with hospitalized people living with cancer, omission was the most reported error (65.1%), and was considered an unintentional error. Reducing dose omission and failures in medication administration in critical groups, such as cancer patients, results in considerable improvements in quality of life, a reduction in readmissions and emergency visits, thus reducing the costs associated with these specific circumstances^(19-23)^.

There is evidence of significant pharmacokinetic variability for many antimicrobials in different patient populations, due to the presence of comorbidities, failing to achieve the therapeutic target and, therefore, antimicrobial pharmacodynamics. Accurate antimicrobial dosing promises to improve patient outcomes. Thus, dose omission in patients with different comorbidities may increase the risk of failure in antimicrobial therapy^(24)^.

A review indicated that comorbidities that lead to organ dysfunction, clinical variables of patients, therapy used (particularly in continuous renal replacement therapy, mechanical ventilation and/or extracorporeal membrane oxygenation) and the inflammatory state of the patient are factors that lead to pathophysiological changes that influence the pharmacokinetic (PK) and pharmacodynamic (PD) parameters of antimicrobials in intensive care^(25)^.

A finding of this study, related to the characteristics of critically ill patients using antimicrobials, was the analysis of the Charlson comorbidity index. The results suggest, although without statistical strength of evidence, that each Charlson unit increases the chance of omitting an antimicrobial dose by 5%. It is suspected that more severe patients have a higher risk of developing HAIs and greater use of antimicrobials and consequently a higher risk of omission. It is expected that this predictor will help identify the complexity of care and the risk of incidents, including omission of antimicrobial doses^(26)^.

For each antimicrobial prescribed in this study, the chance of omission was increased by 51.4%. The number of antimicrobials prescribed was reported as a risk factor for dose omission in another study, which found that an increase in the number of medications prescribed was significantly associated with an increase in omissions; patients who received 20 or more medications were approximately five times more likely to have omissions than patients who received one to four medications (OR 4.99, 95% CI 3.22 to 7.73, p<0.001). Patients taking 15-19 medications were also three times more likely to have an omission compared with those taking one to four medications (OR 3.61, 95% CI 2.86 to 4.56, p<0.001)^(18)^. This study confirms that patients with polypharmacy are a key priority area for improving medication safety.

Polypharmacy is a growing global problem and is related to the increased prevalence of comorbidities, especially in critically ill patients. Therefore, it is important to identify methods to improve avoidable polypharmacy. The minimum number of medications to define polypharmacy in the literature refers to five or more medications taken daily^(27)^. Hyperpolypharmacy, or excessive polypharmacy, is related to the simultaneous use of ten or more medications daily. Patients over 65 years old with multimorbidity are more vulnerable to the adverse effects of polypharmacy and inappropriate prescription and, consequently, have a higher risk of medication-related harms such as omitted antimicrobial doses^(28)^.

Safe medication administration practices are one of the responsibilities of ICU nurses, and double checking has been disseminated as a strategy to prevent medication errors, usually two independent people (manually or electronically) confirm through a signature that a task was performed correctly and in accordance with the procedure^(29)^.

This study found that double checking was performed on 260 prescriptions and that 11 of these had omitted doses, showing that the medication error prevention strategy reduced the risk of omitted doses by 30%. A quality improvement study conducted in an ICU that aimed to reduce the rate of medication errors in the unit to zero included double checking among its interventions and found that medication errors can be reduced through the implementation of several multidisciplinary interventions using human-based approaches and technology^(30)^.

Although double-checking is a widespread practice in intensive care units, a systematic review that assessed the effectiveness of double-checking in reducing medication administration errors concluded that there is insufficient evidence that double-checking versus single-checking of medications is associated with lower error rates. Most comparative studies failed to define or investigate the level of adherence to independent double-checking, further limiting conclusions about its effectiveness in preventing errors. Higher-quality studies are needed to determine whether, and in what context (e.g., type of medication, setting), double-checking produces sufficient benefits in patient safety to warrant the considerable resources required^(31)^.

It is crucial to emphasize that medication preparation and administration are among the main activities that nursing staff perform on a daily basis. Taking into account the lack of standardization of these processes, the high workload and several other activities that must be performed concomitantly with medications, and sometimes hinder this execution, as described by nurses in a qualitative study from the United States. Shift changes were also a factor related to missed doses, probably attributed to the accumulation of functions during this period for the transfer of care to be carried out^(32)^.

For the barriers faced in this process, there are strategies to ensure patient safety, such as: double-checking of prescriptions and medication, creation of medication areas where there are fewer interruptions, and ongoing education actions for the professionals involved^(33)^. In addition, reflection on the use of technologies to facilitate the medication preparation environment, and the nursing environment and workload. Furthermore, as demonstrated in this study, there is still a considerable percentage of errors involving these steps. The lack of human resources may be related to the non-implementation of some safety strategies, such as double-checking, specific professionals responsible only for medication, and an adequate workload so that there is no overload of functions and discontinuity of care^(8)^.

### Study limitations

Although this study was able to establish relationships between the presence of some clinical characteristics and the omission of antimicrobial doses, it was not possible to observe the specific repercussions for the most affected groups. In addition, the lack of checking and the lack of justification in the prescription of the medication may indicate a failure to record the dose and not its omission. Secondary data, due to the collection system and the transport of the Epimed Patient Safety Monitor^®^ to the MV Soul^®^ System, these data may be lost, which caused the exclusion of some prescriptions when this lack of information was relevant to the present study.

### Contributions to the Area

As demonstrated, this study contributes to addressing a relevant topic by exploring the possible repercussions of missed doses and indiscriminate usage of antibiotics, polypharmacy and their clinical correlations. These topics still lack data in literature, and therefore, research such as this offers significant reflections on potential errors in drug prescribing. This contribution is crucial for the advancement of knowledge, providing important reflections that can promote greater safety and improve patient care.

## CONCLUSIONS

It is concluded that the study met its objective by analyzing the rate of omissions of antimicrobial doses in intensive care units and highlights that there may be impairment in antimicrobial pharmacokinetics in patients with comorbidities, subject to polypharmacy and clinical complications, and the omission of antimicrobial doses increases this risk, thus, contributing to therapeutic failure.

Monitoring the dose omission indicator can guide nursing strategies to improve the quality of the process of checking, scheduling and administering antimicrobials, contributing to antimicrobial management and combating antimicrobial resistance and, consequently, improving the quality of care and patient safety.
