# Supplementary material for: Analysis of omission of antimicrobial doses in Intensive Care Units
Source: Rev Bras Enferm. 2024 Dec 16;77(6):e20240102. doi: 10.1590/0034-7167-2024-0102 (PMC11654556; doi:10.1590/0034-7167-2024-0102)
Supplement: Translation

# Translation: Análise de omissão de doses de antimicrobianos em Unidades de Terapia Intensiva

**DOI:** 10.1590/0034-7167-2024-0102pt

**Authors:** Isabelle Ribeiro Machado, Danielle de Mendonça Henrique, Flávia Giron Camerini, Vanessa Galdino de Paula, Cintia Silva Fassarella, Lucas Rodrigo Garcia de Mello

**Affiliations:** I Universidade do Estado do Rio de Janeiro. Rio de Janeiro, Rio de Janeiro, Brasil

**Keywords:** Anti-Infecciosos, Gestão de Antimicrobianos, Unidades de Terapia Intensiva, Erros de medicação, Segurança do Paciente.

## Abstract

**Objetivos:**

analisar a taxa de omissão de dose de antimicrobianos em unidades de terapia intensiva.

**Métodos:**

estudo transversal, realizado entre 1 de março a 30 de setembro de 2023, em unidades de terapia intensiva de um Hospital Universitário no Rio de Janeiro.

**Resultados:**

a amostra foi de 452 prescrições e 1467 doses de antimicrobianos. A taxa de omissão de dose correspondeu a 4,29%. Cada antimicrobiano prescrito aumentou a chance de omissão em 51%. A estratégia de dupla checagem de prescrições contribuiu para prevenção de 30% das omissões de doses de antimicrobianos (p=0,0001).

**Conclusões:**

o monitoramento da omissão de dose de antimicrobianos, pode direcionar ações de enfermagem para melhoria da qualidade e segurança do paciente, contribuindo para prevenção de erros de medicação, gestão de antimicrobianos e combate à resistência antimicrobiana.

## INTRODUÇÃO

Os antimicrobianos são a segunda classe de medicamentos mais utilizados em hospitais e são responsáveis por 20 a 50% das despesas hospitalares com medicamentos^(1)^. Na América Latina, estima-se que os antimicrobianos são utilizados de forma inadequada em 50% dos casos, sendo que o Brasil lidera o *ranking* de consumo de antibióticos, com 22,75 de doses diárias definidas para cada mil habitantes por dia^(2)^.

O uso indiscriminado de antimicrobianos contribui para Resistência Antimicrobiana (RAM) definida como a capacidade de um microrganismo impedir a atuação de um antimicrobiano^(3)^. A infecção por organismos resistentes aumenta a mortalidade, a duração da doença, o tempo de permanência no hospital e, consequentemente, tem um custo muito elevado^(4)^.

As Unidades de Terapia Intensiva (UTI) têm sido consideradas o epicentro das bactérias resistentes a antimicrobianos. Além disso, as UTIs têm enfrentado grandes desafios no combate à RAM, diante da diversidade e disseminação dos microrganismos, em função de fatores contribuintes como: uso de dispositivos invasivos, imunossupressão, idade e o tratamento empírico com antibióticos, bem como o seu uso de forma inadequada e incidência de Infecção Relacionada à Assistência à Saúde (IRAS)^(4)^.

Erros de medicação relacionados à antibioticoterapia se configuram como um problema na UTI, potencializados com aspectos relacionados à fragilidade de conhecimento de farmacologia e processos de trabalho inadequados^(5)^. A omissão de doses, por sua vez, configura-se como um erro de medicação relevante na UTI, devido à ocorrência frequente e ao potencial de dano ao paciente. É caracterizado pela não administração de um medicamento necessário ao paciente, ou de uma ou mais doses prescritas até o próximo horário agendado, excluindo circunstâncias nas quais o paciente se recusa a tomar o medicamento ou quando há decisão médica de não administrar^(6)^.

Um estudo britânico que analisou 90.761 doses de antimicrobianos evidenciou uma taxa de omissão de doses de 7,2%, sendo que 0,9% das doses omitidas foram medicações indisponíveis^(7)^. No Brasil, um estudo realizado na UTI de um Hospital de Ensino do Distrito Federal, coletou uma amostra com 711 doses de antimicrobianos e 48 doses foram omitidas, correspondendo a uma de taxa de erro na administração de antimicrobianos por omissão de doses de 6,75%^(8)^.

Pacientes internados em UTI apresentam cinco a dez vezes maior probabilidade de manifestar IRAS^(9)^. Tal fato reflete uma polifarmácia antimicrobiana, com prescrições de antimicrobianos de última geração, contribuindo para o uso irracional e para a consequente resistência antimicrobiana, podendo potencializar o risco de omissões de doses de um antimicrobiano, causando danos à segurança do paciente^(10)^.

Um estudo na Etiópia identificou a prescrição de antimicrobianos em 80% dos pacientes internados em enfermarias e 100% dos pacientes no UTI. Todavia, menos de 1% desses pacientes realizaram exames microbiológicos antes do início tratamento, com diagnóstico preciso de infecção por microrganismos patogênicos, corroborando para o uso irracional de antimicrobianos e resistência antimicrobiana^(11)^.

A gestão de antimicrobianos tem sido uma estratégia fundamental para mitigar a RAM e a omissão de doses^(12)^. Ela envolve um conjunto de ações destinadas ao controle do uso desses medicamentos desde o diagnóstico, seleção, prescrição e a dispensação adequada às boas práticas de diluição, conservação e administração, além da auditoria e do monitoramento das prescrições, da educação de profissionais e pacientes, do monitoramento do programa até a adoção de medidas intervencionistas, assegurando resultados terapêuticos ótimos com mínimo risco potencial^(13)^.

A participação efetiva dos enfermeiros nos programas de gestão de antimicrobianos, entre outras ações, pode contribuir para mitigar a omissão de dose de antimicrobianos e reduzir desfechos desfavoráveis como taxas de mortalidade global e específica relacionada a microrganismos multirresistentes, tempo de hospitalização, incidência de IRAS, readmissão relacionada a diagnósticos infecciosos e taxas de reações adversas aos antibióticos, refletindo na melhoria de qualidade da assistência e segurança do paciente^(12)^.

## OBJETIVOS

Analisar a taxa de omissão de dose de antimicrobianos em unidades de terapia intensiva.

## MÉTODOS

### Aspectos éticos

O estudo foi conduzido de acordo com as diretrizes de ética nacionais e internacionais e aprovado pelo Comitê de Ética em Pesquisa (CEP) da Universidade do Estado do Rio de Janeiro (UERJ), de acordo com a Resolução 466/12, cujo parecer está anexado à presente submissão.

O Termo de Consentimento Livre e Esclarecido (TCLE) foi dispensado uma vez que o levantamento e coleta de dados foi realizada pelo acesso a documentos como prontuários e prescrições através do número do registro, que serve apenas para validar a individualidade da informação. Além disso, a confidencialidade da identificação pessoal dos pacientes foi garantida pelo pesquisador principal.

### Desenho, período e local do estudo

Trata-se de um estudo transversal, seguindo os vinte e dois passos das recomendações do *Strengthening the Reporting of Observational Studies in Epidemiology* (STROBE)^(14)^.

O Cenário do estudo foi composto por uma unidade de terapia intensiva geral e uma unidade cardiointensiva de um Hospital Universitário do Sistema Único de Saúde (SUS), localizado no município do Rio de Janeiro - RJ. Cada uma das unidades possui 10 leitos de internação e, frequentemente, mantém uma taxa de ocupação de 100%. A coleta de dados foi realizada entre 01 de março a 30 setembro de 2023.

### População, amostra, critérios de inclusão e exclusão

A população do estudo foi composta de prescrições de pacientes internados nas duas unidades de terapia intensiva no período de 12 meses, contabilizando 7920 prescrições. Para o cálculo de amostragem probabilística, optou-se pela amostra aleatória simples, em que todos os elementos da população têm igual probabilidade de pertencer à amostra realizada pela calculadora Epiinfo^®^.

Considerando a população descrita, este estudo optou por atribuir como percentual máximo a prevalência de 47,9% de erros relacionados à omissão de doses, conforme especificado pelo Instituto para Práticas Seguras no Uso de Medicamentos (ISMP)^(5)^. Foi atribuído o índice de confiança de 97%, dessa forma, a amostra calculada foi de 444 prescrições.

Para critério de inclusão, considerou-se prescrições de pacientes acima de 18 anos internados nas unidades de terapia intensiva, com prescrição de antimicrobiano por via endovenosa. Foram excluídas as prescrições em que as doses foram omitidas por recusa do paciente ou por suspensão médica, visto que esses casos não caracterizam omissão de dose.

### Protocolo do estudo

As informações de interesse foram coletadas das prescrições e registros de enfermagem impressos e do prontuário eletrônico, a partir de um formulário cujas variáveis foram ancoradas no boletim de Prevenção de erros de medicação por omissão do ISMP^(5)^ e inseridas no *software* Epimed Monitor Segurança do Paciente^®^ para gestão de incidentes (*Epimed Solutions* ^®^, Rio de Janeiro), que é integrado ao Sistema MV Soul^®^ (prontuário eletrônico utilizado no hospital do estudo).

Foram incluídas as variáveis relacionadas ao perfil clínico do paciente: idade, gênero, diagnóstico, data da internação e desfecho (alta, óbito e transferência), preditores de gravidade e mortalidade. Assim, as variáveis relacionadas à omissão de doses foram coletadas a partir das prescrições médicas do dia anterior à coleta, do número total de doses de antimicrobianos prescritos e da dupla checagem.

Destaca-se, neste estudo, que a omissão de dose foi computada a partir da análise das prescrições e registros de enfermagem de não administração do antimicrobiano, de modo que as doses que não foram checadas ou que foram rodeladas sem justificativa foram consideradas omitidas. Além disso, as unidades do estudo adotam a dupla checagem como uma ferramenta de segurança medicamentosa, sendo possível de ser auditada, uma vez que o enfermeiro registra na prescrição que a dupla checagem foi realizada e incluindo data e carimbo.

### Análise dos resultados e estatística

Os dados foram organizados e tabulados em uma planilha eletrônica *(Microsoft Excel* ^®^). As variáveis numéricas foram descritas como: média, desvio padrão, mediana, primeiro e terceiro quartis, já as variáveis categóricas, como: frequência absoluta e relativa. O indicador de taxa de omissão de doses foi calculado pela divisão entre o número de doses omitidas e número de doses totais de antimicrobianos prescritos.

A relação da taxa de omissão com as variáveis de interesse (local, sexo, dupla checagem, comorbidades, idade, número de antibióticos, preditores de gravidade e mortalidade *Charlson e Simplified Acute Physiology Score* 3 [SAPS3]) foi avaliada com modelos de regressão logística. Intervalos de confiança 95% foram calculados para as taxas de omissão e razões de chances estimadas. Todas as análises foram conduzidas no pacote estatístico R versão 4.3.1

## RESULTADOS

Foram analisadas 452 prescrições de 94 pacientes internados nas Unidades de Terapia Intensiva. A taxa de omissão de doses calculada, neste estudo, foi 4,29%, considerando o total de 1467 doses de antimicrobianos analisadas e 63 doses omitidas, ou seja, cerca de 4 doses de antimicrobianos foram omitidas em cada 100 prescrições.

A média de doses de antimicrobianos prescritos por paciente foi 15,61 e mediana 7,5. A média de idade dos pacientes foi de 59 anos, sendo o primeiro quartil 54 anos e o terceiro quartil 69 anos, evidenciando que a metade dos sujeitos está concentrada nessa faixa etária.

A Tabela 1 apresenta a probabilidade de omissão de dose de antimicrobianos em função das variáveis relacionadas aos pacientes. Os resultados apontaram significância estatística, com valores-p menores que 0,05 quanto ao local de internação, sexo e na presença das comorbidades, câncer, HAS, obesidade, imunossupressão, insuficiência cardíaca congestiva (ICC).

**Tabela 1:** Probabilidade de omissão de doses de antimicrobianos relacionadas às características dos pacientes, Rio de Janeiro, Rio de Janeiro, Brasil, 2023

Preditor	Nível	Prevalência	Probabilidade	IC	Valor de *p*
Local de Internação	UTI Geral	51,06%	5,39%	(4,11 - 7,05)	0,026
	Cardiointensiva	48,94%	2,96%	(1,82 - 4,78)	
Sexo	Feminino	48,94%	3,12%	(2,06 - 4,69)	0,013
	Masculino	51,06%	5,78%	(4,33 - 7,68)	
Comorbidades					
Câncer	Não	72,34%	2,60%	(1,76 - 3,81)	<0,001
	Sim	27,66%	8,13%	(6,05 - 10,86)	
DM	Não	41,49%	5,02%	(3,66 - 6,85)	0,332
	Sim	58,51%	3,97%	(2,77 - 5,66)	
HAS	Não	24,47%	7,61%	(5,48 - 10,46)	<0,001
	Sim	75,53%	3,14%	(2,23 - 4,40)	
Obesidade	Não	94,68%	4,73%	(3,73 - 5,99)	0,049
	Sim	5,32%	1,06%	(0,15 - 7,16)	
Imunossupressão	Não	71,28%	5,32%	(4,12 -6,85)	0,01
	Sim	28,72%	2,41%	(1,30 - 4,42)	
Asma	Não	93,62%	4,64%	(3,65 - 5,87)	0,17
	Sim	6,38%	1,52%	(0,21 - 9,98)	
ICC	Não	56,38%	6,23%	(4,81 - 8,03)	<0,001
	Sim	43,62%	1,88%	(1,05 - 3,37)	
Fibrilação Atrial Crônica	Não	78,26%	4,75%	(3,67 - 6,14)	0,644
	Sim	21,74%	4,10%	(2,29 - 7,26)	

*IC - Intervalo de Confiança 95%.*

Quanto ao local de internação, observou-se que 48 pacientes (51,06%) estavam internados na unidade de terapia intensiva geral e 46 (48,94%) na unidade cardiointensiva, a UTI geral apresentou 5,39% de probabilidade de ocorrência de omissão de doses de antimicrobianos (p-valor: 0,026). Houve um equilíbrio na prevalência do sexo feminino 46 (48,94%) e masculino 48 (51,06%), porém o sexo masculino teve maior probabilidade de omissão (5,78%) do que o feminino (3,12%).

Dentre as comorbidades com maior prevalência destacam-se hipertensão arterial sistêmica (75,53%), diabetes mellitus (58,51%) e insuficiência cardíaca congestiva (43,63%). Porém, em pacientes diabéticos não houve significância para distinguir as taxas avaliadas. A prevalência do câncer foi 27,66% e pacientes com essa doença se destacaram por apresentar maior probabilidade de omissão de doses de antimicrobianos (8,13%) com significância estatística relevante (p-valor <0,001).

A Tabela 2 apresenta a probabilidade de omissão de doses a partir dos preditores *Charlson e Simplified Acute Physiology Score 3* (SAPS3), além da quantidade de antimicrobianos prescritos, representando aspectos de gravidade do paciente da terapia intensiva.

**Tabela 2:** Probabilidade da ocorrência de omissão de doses de antimicrobianos em pacientes críticos, Rio de Janeiro, Rio de Janeiro, Brasil, 2023

Preditores de Gravidade	RP	IC	Valor de *p*
*Charlson*	1.05	(0.92-1.18)	0.44
*SAPS3*	0.99	(0.97-1.01)	0.60
Número de Antimicrobianos prescritos	1.51	(1.21-1.87)	<0.001

*RP - Razão de Prevalência; IC - Intervalo de Confiança 95%.*

Foi identificado significância estatística (p<0.001) no que se refere ao quantitativo de antimicrobianos por prescrição. Cada antimicrobiano prescrito aumenta a chance de omissão em 51%. Quanto aos preditores de gravidade, ainda que não tenha significância, pode-se observar que cada unidade do escore de comorbidade de *Charlson* aumenta a chance de omissão de dose de antimicrobianos em 5%.

A Tabela 3 apresenta a probabilidade de ocorrência de omissão de doses de antimicrobianos em função da realização da dupla checagem, uma estratégia de segurança do paciente realizada por enfermeiros voltada para prevenção de erros de medicação e gestão de antimicrobianos.

**Tabela 3:** Associação das omissões de doses de antimicrobianos com a estratégia de segurança dupla checagem de prescrições, Rio de Janeiro, Rio de Janeiro, Brasil, 2023

		Omissão de Doses		RP	IC	Valor de *p*
Exposição		Sim	Não			
Dupla Checagem	Sim	11	249	0.30	0.15-0.59	0.0001
	Não	27	165			
Total		38	414			

*RP - Razão de Prevalência; IC - Intervalo de Confiança 95%.*

A dupla checagem foi realizada em 260 prescrições e em 11 delas ocorreu omissão de doses de antimicrobianos, com significância estatística (p valor 0,0001). Ao calcular razão de prevalência, foi evidenciado que a dupla checagem pode prevenir em 30% a omissão de doses de antimicrobianos.

## DISCUSSÃO

Omissões de doses de antimicrobianos são erros de medicações, com um impacto desfavorável para os pacientes críticos, sendo considerado um incidente que resulta em aumento no tempo de internação, no risco de sepse e da mortalidade^(15)^. Além disso, os antimicrobianos são administrados em ciclos relativamente curtos, desse modo, a omissão de doses pode levar a concentrações elevadas e contribuir para resistência antimicrobiana^(16)^.

As taxas de omissões relatadas por estudos anteriores são muito variáveis, possivelmente devido às diversas definições e sistemas de classificação de omissão. Portanto, foi difícil traçar aspectos comparativos visto que alguns estudos analisam “doses”, equanto outros verificam “pacientes”. Contudo, o presente estudo revelou uma taxa de omissão de dose de antimicrobiano de 4,29%. Outro estudo nacional realizado em uma unidade de terapia intensiva analisou uma amostra de 711 doses de antimicrobianos, em que ocorreram 48 omissões, correspondendo a uma taxa de omissão 6,75%^(7)^. Outros estudos sobre a temática, variaram de 0,9% no Reino Unido e 28,8% em Zâmbia^(8,17)^.

Para além da taxa de omissões de doses, as características de grupos de pacientes podem ser destacadas para serem priorizadas nas estratégias de prevenção de omissão de doses de antimicrobianos^(17)^. Ao associar as características dos pacientes críticos em uso de antimicrobianos com a omissão de doses dos referidos medicamentos, este estudo observou uma proporção mais elevada de omissões de antimicrobianos em pacientes do sexo masculino (48,94% vs. 51,06%, [p=0,013]). Esse achado alinha-se com outra pesquisa que também identificou uma prevalência maior de omissões no sexo masculino (51,4% vs. 35,9%, [p=0,075])^(18)^.

As comorbidades mais prevalentes com maior probabilidade de omissão de antimicrobianos foram entre os pacientes portadores hipertensão arterial sistêmica, insuficiência cardíaca congestiva e câncer. Considerando este um importante dado epidemiológico, em um estudo sobre discrepância medicamentosa realizado com pessoas hospitalizadas vivendo com câncer, a omissão foi o erro mais relatado (65.1%), sendo considerado erro não intencional. Reduzir a omissão de doses e falhas na administração de medicamentos em grupos críticos, a exemplo dos pacientes com câncer, resulta em melhorias consideráveis na qualidade de vida, uma redução nas readmissões e visitas de emergência, diminuindo, assim, os custos associados a essas circunstâncias particulares^(19-23)^.

Há comprovação de uma variabilidade farmacocinética significativa para muitos antimicrobianos em diferentes populações de pacientes, devido à presença de comorbidades, falhando em atingir a meta terapêutica e, portanto, a farmacodinâmica antimicrobiana. A dosagem antimicrobiana realizada de forma precisa promete melhorar os resultados dos pacientes. Assim, a omissão de doses em pacientes com diferentes comorbidades pode potencializar o risco de falha na terapêutica antimicrobiana^(24)^.

Uma revisão apontou que comorbidades que levam a disfunções orgânicas, variáveis clínicas dos pacientes, terapêutica utilizada (em particular, a terapia renal substitutiva contínua, ventilação mecânica e/ou oxigenação por membrana extracorpórea) e o estado inflamatório do paciente são fatores que levam a alterações fisiopatológicas que influenciam a farmacocinética (PK) e farmacodinâmica (PD), parâmetros de antimicrobianos em terapia intensiva^(25)^.

Um achado deste estudo, relacionado às características do paciente crítico em uso de antimicrobiano, foi a análise do índice de comorbidade de *Charlson*. Os resultados sugerem, ainda sem força de evidência estatística, que cada unidade de *Charlson* aumenta a chance de omissão de dose de antimicrobianos em 5%. Suspeita-se que os pacientes mais graves têm maior risco de desenvolver IRAS e uso maior de antimicrobianos e, consequentemente, maior risco de omissão. Espera-se que este preditor auxilie na identificação da complexidade do cuidado e do risco da ocorrência de incidentes, incluindo a omissão de doses de antimicrobianos^(26)^.

A cada antimicrobiano prescrito neste estudo, a chance de omissão foi aumentada em 51.4%. O quantitativo de antimicrobianos prescrito foi relatado como fator de risco para omissão de dose em outra pesquisa, a qual identificou o aumento do número de medicamentos prescritos como significativamente associado ao aumento nas omissões; os pacientes que receberam 20 ou mais medicamentos tiveram cerca de cinco vezes mais probabilidade de ter omissões do que os pacientes que receberam um a quatro medicamentos (OR 4,99, IC 95% 3,22 a 7,73, p<0,001). Os pacientes que tomavam 15-19 medicamentos também tinham uma probabilidade três vezes maior de ter uma omissão em comparação com aqueles que tomavam de um a quatro medicamentos (OR 3,61, IC 95% 2,86 a 4,56, p<0,001)^(18)^.

Este estudo confirma que os pacientes com polifarmácia são uma das principais áreas para a melhoria da segurança dos medicamentos. A polifarmácia é um problema global crescente e se relaciona com aumento da prevalência de comorbidades, em especial de pacientes críticos. Portanto, é importante identificar métodos para melhorar a polifarmácia evitável. O mínimo de medicamentos para definir polifarmácia na literatura se refere a cinco ou mais medicamentos tomados diariamente^(27)^. A Hiperpolifarmácia, ou polifarmácia excessiva, relaciona-se ao uso simultâneo de dez ou mais medicamentos diários. Pacientes com mais de 65 anos com multimorbidade são mais vulneráveis aos efeitos adversos da polifarmácia e da prescrição inadequada e, consequentemente, apresentam maior risco de danos relacionados à medicação como a omissão de dose de antimicrobianos^(28)^.

Práticas seguras de administração de medicamentos são uma das responsabilidades dos enfermeiros da UTI, a realização da dupla checagem tem sido difundida como uma estratégia para prevenção de erros de medicação. Usualmente duas pessoas independentes (manualmente ou eletronicamente) confirmam por assinatura que uma tarefa foi executada corretamente e de acordo com o procedimento^(29)^.

Este estudo apontou que a dupla checagem foi realizada em 260 prescrições e em 11 delas ocorreu omissão de doses, evidenciando que a estratégia de prevenção de erros de medicação reduziu o risco de omissão de doses em 30%. Um estudo de melhoria de qualidade realizada em uma UTI, cujo objetivo foi zerar a taxa de erros de medicação na unidade, incluiu a dupla checagem entre suas intervenções e identificou que os erros de medicação podem ser diminuídos através da implementação de várias intervenções multiprofissionais utilizando abordagens baseadas em humanos e tecnologia^(30)^.

Ainda que seja uma prática difundida em unidades de terapia intensiva, uma revisão sistemática, a qual objetiva a eficácia da dupla checagem na redução de erros na administração de medicamentos, conclui que não há evidências suficientes de que a dupla checagem versus a verificação única de medicamentos esteja associada a taxas mais baixas de erros. A maioria dos estudos comparativos não conseguiu definir ou investigar o nível de adesão à dupla verificação independente, limitando ainda mais as conclusões sobre a eficácia na prevenção de erros. São necessários estudos de maior qualidade para determinar se, e em que contexto (por exemplo, tipo de medicamento, ambiente), a dupla verificação produz benefícios suficientes na segurança do paciente para garantir os recursos consideráveis necessários^(31)^.

É crucial salientar que a preparação e administração de medicamentos estão entre uma das principais atividades que a equipe de enfermagem exerce diariamente, sobretudo levando em consideração que a falta de padronização desses processos, a alta carga de trabalho e diversas outras atividades que devem ser executadas concomitante às medicações, por vezes, atrapalham essa execução, conforme descrito por enfermeiros em estudo qualitativo dos Estados Unidos. A mudança de turno também foi um fator relacionado à omissão de doses, provavelmente atribuído ao acúmulo de funções nesse período para que a transferência de cuidados seja realizada^(32)^.

Para as barreiras enfrentadas nesse processo, existem estratégias para garantir a segurança do paciente como: dupla-checagem da prescrição e medicação, criação de áreas de medicação onde há menos interrupções, ações de educação continuada dos profissionais envolvidos^(33)^. Além disso, destaca-se a reflexão sobre o uso de tecnologias na facilitação do ambiente de preparo de medicações, o ambiente e a carga de trabalho da enfermagem. Ademais, como demonstrado neste estudo, ainda há um percentual considerável de erros envolvendo estas etapas. A falta de recursos humanos pode estar relacionada à não execução de algumas estratégias de segurança como a dupla checagem, profissionais específicos responsáveis somente pela medicação e uma carga de trabalho adequada para que não haja sobrecarga de funções e descontinuidade do cuidado^(8)^.

### Limitações do estudo

Embora este estudo tenha conseguido estabelecer relações entre a presença de algumas características clínicas e a omissão de doses de antimicrobianos, não foi possível observar as repercussões específicas para os grupos mais afetados. Além disso, a não checagem e a falta de justificativa na prescrição do medicamento pode indicar uma falha de registro da dose e não sua omissão. Os dados secundários, devido ao sistema de coleta e o transporte do Epimed Monitor Segurança do Paciente^®^ para o Sistema MV Soul^®^, podem sofrer perdas, o que causou a exclusão de algumas prescrições quando a ausência de informação era relevante ao presente estudo.

### Contribuições para a Área

Conforme demonstrado, este estudo contribui para a abordagem de uma temática relevante ao explorar as possíveis repercussões da omissão de doses, o uso indiscriminado de antibióticos, a polifarmácia e suas correlações clínicas. Esses tópicos ainda carecem de dados na literatura, e, portanto, pesquisas como esta oferecem reflexões significativas sobre os potenciais erros na prescrição medicamentosa. Tal contribuição é crucial para o avanço do conhecimento, fornecendo reflexões importantes que podem promover maior segurança e aprimoramento da assistência ao paciente.

## CONCLUSÕES

Conclui-se que o estudo respondeu ao objetivo, ao analisar a taxa de omissões de doses de antimicrobianos nas unidades de terapia intensiva. Destaca-se que pode haver comprometimento na farmacocinética antimicrobiana nos pacientes com comorbidades, sujeitos à polifarmácia e à complicações clínicas, nesse sentido, a omissão de doses de antimicrobianos potencializa o risco, contribuindo portanto para falha na terapêutica.

O monitoramento do indicador de omissão de dose pode direcionar estratégias de enfermagem para melhoria da qualidade no processo de checagem, aprazamento e administração de antimicrobianos, contribuindo para a gestão, para o combate à resistência antimicrobiana e, consequentemente, aprimorando a qualidade da assistência e segurança do paciente.
